# Wunderlich Syndrome With a Myriad of Presentations: A Case Series

**DOI:** 10.7759/cureus.70948

**Published:** 2024-10-06

**Authors:** Karthik M Chavannavar, Sriram Krishnamoorthy, Natarajan Kumaresan, Chandru T

**Affiliations:** 1 Urology, Sri Ramachandra Institute of Higher Education and Research, Chennai, IND

**Keywords:** interventional radiology guided embolization, renal angiomyolipoma, tuberous sclerosis complex (tsc), urology emergency, wunderlich syndrome

## Abstract

Wunderlich syndrome (WS), characterized by spontaneous nontraumatic renal or perinephric hemorrhage, presents a significant diagnostic challenge due to its varied causes and clinical manifestations. Despite its historically high case fatality rate, prompt and accurate diagnosis combined with a multidisciplinary treatment approach has been shown to significantly improve patient outcomes. This case series discusses three patients with diverse presentations of WS, each managed with tailored therapeutic strategies involving a combination of conservative management, super selective renal artery embolization, and surgical interventions such as nephrectomy. The successful outcomes in these cases underscore the importance of high clinical suspicion, early diagnosis, and comprehensive management to mitigate the syndrome's potentially fatal consequences. Subcapsular hematomas, typically self-limiting, highlight the role of conservative management in renal preservation. This series reinforces that timely and appropriate intervention can transform the prognosis of WS from lethal to manageable.

## Introduction

Wunderlich syndrome (WS) is defined as spontaneous, nontraumatic renal or perinephric hemorrhage [1]. It presents as Lenk’s triad: acute flank pain, mass, and hypovolemic shock [2]. However, this triad is seen in only a quarter of these patients [3]. WS can be due to various causes, like renal tumors, inflammatory causes, or arteriovenous malformations. WS is uncommon. Renal angiomyolipoma was described by Grawitz in 1900 as a benign entity made up of dysmorphic blood vessels, smooth muscle, and adipose tissue [4]. These tumors are associated with genetic syndromes such as tuberous sclerosis (TSC) or lymphangioleiomyomatosis (LAM) or may occur sporadically. It is most commonly seen in female sex and peaks in the fourth to fifth decade [5]. Patients with TSC have 55% to 90% prevalence and present earlier than the sporadic cases [6]. However, the disease can present in varied ways. If not picked up and treated appropriately in time, the outcomes are usually fatal (7.4%-28%) [7]. We present our experience of three cases with different symptomatology and modes of management of these cases with positive outcomes.

## Case presentation

Case 1

**Figure 1:**
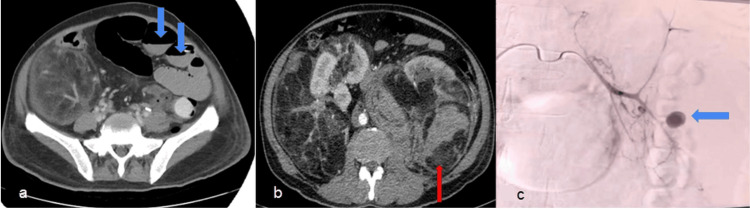
Computed tomography of the abdomen and renal angiogram images AML: Angiomyolipoma (a) Computed tomography abdomen showing the dilated bowel loops (blue arrow) with multiple air-fluid levels indicating subacute intestinal obstruction. (b) Computed tomography of the abdomen showing bilateral renal AML, right inferior pole renal angiomyolipoma, and left renal angiomyolipoma replacing the kidney in its entirety with hematoma in the lower pole (red arrow). (c) Pseudoaneurysm of the left renal artery (blue arrow). Super selective renal artery embolization was done

A gentleman in his mid-50s came to the emergency room (ER) with acute generalized abdominal pain and vomiting. He was a known case of tuberous sclerosis. He had a heart rate of 120 beats per minute and blood pressure of 70/50 mmHg at presentation. Clinically, he had a distended abdomen with generalized tenderness, and no bowel sounds were heard. Initial resuscitative measures were taken, and the patient was stabilized. The hematology and blood biochemistry investigations are given in Table 1. The ultrasound showed dilated bowel loops and partially visualized the left kidney with multiple hyperechoic regions replacing the entire kidney. The right kidney was visualized with multiple hyperechoic lesions. A contrast-enhanced computed tomography (Figures 1a-1b) was done, which showed an 8.1 x 9.1 x 10.8 cm right renal AML and a 14.8 x 13.2 x 18.4 cm left renal AML with a hyperdense collection measuring 85 cc in the lower pole on the left side and multiple saccular aneurysms in the segmental branches of the midpole. It also showed dilated small bowel loops, suggestive of small bowel obstruction, possibly secondary to large retroperitoneal mass and collection. Based on the presentation and dilated bowel loops in the contrast-enhanced computed tomography, the other differential diagnosis was acute/subacute intestinal obstruction.

**Table 1:** Laboratory investigations along with reference values gm/dl: grams per deciliter; cells/mm^3^: cells per cubic millimeter; mg/dl: milligrams per deciliter; WBC: white blood cell Reference range: from our institutional laboratory

Investigation (unit)	Case 1	Case 2	Case 3	Reference range
Hemoglobin (gm/dl)	7	7.5	10.1	12-18
WBC count (cells/mm^3 ^)	21000	18030	9200	4000-11000
Platelets (cells/mm^3 ^)	100000	80000	280000	150000-400000
Serum creatinine (mg/dl)	0.9	0.8	0.8	0.8-1.2

A multidisciplinary team involving a urologist, critical care physician, surgical gastroenterologist, interventional radiologists, and a team of nurses was involved. A nasogastric tube was inserted to decompress the stomach and bowel loops, and crystalloid fluids along with packed cells were transfused. He was put on invasive ventilation and underwent an emergency bilateral selective renal artery embolization (Figure 1c). His condition was stabilized. Subacute intestinal obstruction was treated with conservative management. We counseled the patient and performed a left open nephrectomy. He recovered well. He has been on follow-up for the right renal AML for the past three months. He has been advised yearly follow-up with MRI. The histopathology of the left nephrectomy specimen was renal AML.

Case 2

**Figure 2:**
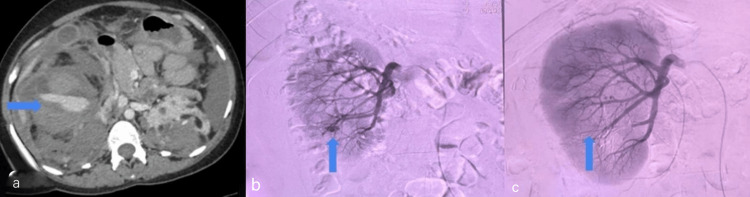
Computed tomography of the abdomen and renal angiogram images AML: Angiomyolipoma (a:) Bilateral renal AML with active bleeding from the largest lesion of the right kidney (blue arrow) causing hemo-retroperitoneum and moderate hemoperitoneum. (b) Right renal artery angiography showed a pseudoaneurysm (blue arrow) with active blush in the interpole and lower pole. (c) Post superselective renal artery embolization (blue arrow)

A lady in her early 30s came to the ER with complaints of acute-onset pain in the right flank, altered sensorium, and hypovolemic shock. She was a known case of tuberous sclerosis. She was one-week postpartum at the time of presentation. At the presentation, she was drowsy and had hypotension (70/40 mmHg) with tachycardia (126 beats/min). On clinical examination, she had pallor and generalized tenderness of the abdomen on palpation. She was resuscitated and stabilized in the ER. She has a history of subependymal giant cell astrocytoma, which was treated surgically as she had secondary obstructive hydrocephalus. She was also on mTOR inhibitors as a medical line of management for the tuberous sclerosis complex. The hematology and blood biochemistry investigations are given in Table 1. The ultrasound showed bilateral hyperechoic lesions in the kidneys with areas of possible hemorrhage. A contrast-enhanced computed tomogram (Figure 2a)of the abdomen showed bilateral renal AML with active bleeding from the largest lesion (11.6 x 8.7 x 10.0 cm) of the right kidney, causing hemo-retroperitoneum and moderate hemoperitoneum. Contrast-filled, ill-defined lesions in the lower pole of the right kidney were noted, suggestive of pseudoaneurysm. Hepatic AMLs, adrenal myelolipoma, and ovarian dermoids were also noted.

A multidisciplinary team involving a urologist, critical care physician, obstetrician, interventional radiologists, and a team of nurses was involved. She was put on invasive ventilation with ionotropic supports and also was transfused with packed cells. She underwent bilateral superselective renal artery embolization (Figures 2b-2c), and intraoperative two units of packed cell transfusions were given. Post-embolization, she was stable and underwent a right open nephrectomy after two weeks. She recovered well. The initial size of the left renal mass was 3.7 x 3.3 x 3.5 cm. Based on the guidelines of the International Tuberous Sclerosis Complex Consensus, the patient has been advised with follow-up by yearly MRI. However, she has also been advised with a follow-up every six months with ultrasound. The histopathology of the right nephrectomy specimen came out as AML.

Case 3

**Figure 3:**
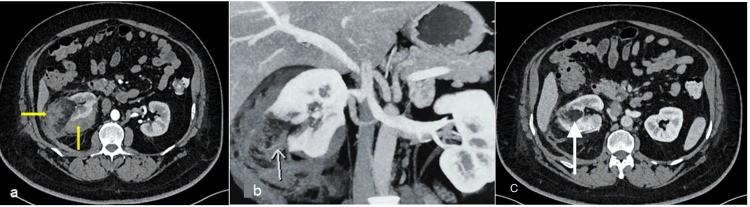
Computed tomography images of the abdomen (a) A subcapsular hematoma in the right kidney with heterodense density lesion seen arising from the interpole of the right kidney (yellow arrows). (b) At 2 weeks, the subcapsular hematoma is seen to be resolving with right renal angiomyolipoma (white arrow). (c) At eight weeks, there is complete resolution of the subcapsular hematoma (white arrow)

A gentleman in his early 50s came to the ER with complaints of right loin pain for one day. He was a known case of type 2 diabetes mellitus. On examination, he had hypotension (80/40 mm Hg) and tachycardia (116 beats/min). Initial resuscitation was done, and his blood pressure was stabilized. The hematology and blood biochemistry investigations are given in Table 1. A bedside ultrasound was performed, which showed a collection in the perinephric region. A contrast-enhanced computed tomogram (Figure 3a) was done, which showed a 4.0 x 4.0 x 3.2 cm well-defined heterogeneous exophytic lesion with a predominant macroscopic fat component arising from the interpole of the right kidney along with a collection of 120 cc in the perinephric region. Strict immobilization and bed rest was advised along with other supportive measures and antibiotics to prevent abscess formation/secondary infection. Repeat imaging was done at two weeks to assess the hematoma (Figure 3b). The collection was resolved completely over eight weeks (Figure 3c). He underwent a partial nephrectomy for the right renal AML after 10 weeks. He recovered well. He has been on follow-up for six months. He has been advised follow-up with computed tomography after one year. The histopathology of right partial nephrectomy specimen came out as AML.

The summary of presentations of all the three cases is given in Table 2.

**Table 2:** Varied presentations of Wunderlich syndrome with management

S No	Age	Sex	Mode of presentation	Comorbidities	Sepsis	Initial treatment	Definitive treatment	Outcome
1	55Y	M	Subacute intestinal obstruction	Tuberous sclerosis, systemic hypertension	Present	Conservative management of obstruction, left super selective renal artery embolization	Left open nephrectomy	Recovered
2	32Y	F	Immediate postpartum in hypovolemic shock	Tuberous sclerosis	Present	Resuscitation, bilateral super selective renal artery embolization	Right open nephrectomy	Recovered
3	51Y	M	Loin pain, hypotension	Type II diabetes mellitus	Absent	Strict immobilization, resuscitation, conservative management	Right partial nephrectomy	Recovered

## Discussion

Spontaneous, nontraumatic, renal, and perinephric bleeding was first documented by Bonet et al. [8] in the year 1679. Later, this condition was described by Wunderlich et al. [9] in 1856. Although Lenk’s triad has been described, there can be varied presentations. Lenk’s triad is seen only in one-third of these patients. It may mimic various other acute abdominal conditions. WS is a perilous and sometimes fatal complication of renal AMLs. Few AMLs are hormone-sensitive (with ~25% of them having progesterone and estrogen receptors), they are therefore more likely to increase in size during pregnancy and rupture [10]. For that reason, many urologists favor close observation of women with known AMLs who are of childbearing age or are pregnant.

At presentation, these patients need resuscitation in case of active bleeding and hypovolemic shock. After stabilization, ultrasound is an effective tool in deciding the line of management. Contrast-enhanced computed tomography is 100% sensitive to finding the underlying cause of bleeding. It has higher sensitivity and specificity than ultrasound in diagnosing the underlying mass [11]. If there is an active blush, saccular outpouching with fat density on the contrast computed tomogram, it is suggestive of pseudoaneurysm, bleeding renal AML. Magnetic resonance imaging is an alternative to computed tomography and has comparable sensitivity and specificity.

Brkovic et al. suggested that angiography is mandatory when computed tomography fails to find the underlying cause [12]. However, their series had a high incidence of polyarteritis nodosa (PAN). Mukamel et al. also insisted on the role of angiography [13].

A meta-analysis done by Zhang et al. published that in 61.5% of cases, etiology was tumors (31.5% malignant and 29.7% benign), vascular disease accounted for 17% of cases, infection for 2.4%, and in 6.7% the cause was idiopathic [11]. Cozzoli et al. have reported this condition even during pregnancy [14].

The prognosis of WS is largely contingent upon the rapidity of diagnosis and the appropriateness of the therapeutic intervention. The probability of bleeding in renal AMLs less than 4 cm is approximately 13% and can go up to as high as 51% if the lesion is above 4 cm in size [15]. Our case series demonstrated favorable outcomes in patients who received prompt and appropriate care. This underscores the importance of early recognition and intervention, which can significantly reduce morbidity and mortality. The literature review further supports that while the overall prognosis can be good, delays in treatment or misdiagnosis can lead to adverse outcomes.

Management strategies also exhibit some variation across different case series. López Cubillana et al. highlighted a conservative approach in all of their cases [16]. We also managed a patient with conservative management successfully.

mTOR inhibitors

Patients with TSC and LAM can now quickly get mTOR inhibitors due to the discovery of TSC1 and TSC2 mutations and the availability of an approved medication that targets the mTOR pathway. These medications help to reverse pre-existing lesions and stop the growth of new tumors. The first mTOR inhibitor to be tried for the treatment of hereditary AML was sirolimus, commonly referred to as rapamycin. It was first used as an immunosuppressive drug for organ transplant recipients. In these early investigations, sirolimus was generally well-tolerated; the most frequent adverse effects reported were dyslipidemia, proteinuria, mouth ulcers, and skin lesions. It can now be used to treat LAM. The most researched rapamycin derivative and mTOR inhibitor is everolimus. At present, there is approval from the European Medicines Agency and the Food and Drug Administration(FDA) for this drug in the setting of TSC.

Ablation

Comparing the available therapeutic approaches, percutaneous radiofrequency ablation and cryoablation seem like appealing substitutes for embolization or surgery. Various series show good results with little adverse reactions, few repeat treatments, and no recurrences. However, instances of using these minimally invasive procedures are confined to very small cohorts and asymptomatic lesions.

Active surveillance

After the first diagnostic assessments are finished and there are no therapeutic indications, active monitoring should be employed to track the growth of new tumors as well as the progression of established ones. For small, single lesions, it appears that annual imaging is appropriate. For hereditary AML, the International Tuberous Sclerosis Complex Consensus supports the use of MRI, because of its greater sensitivity in the detection of adipose tissue [17].

**Figure 4:**
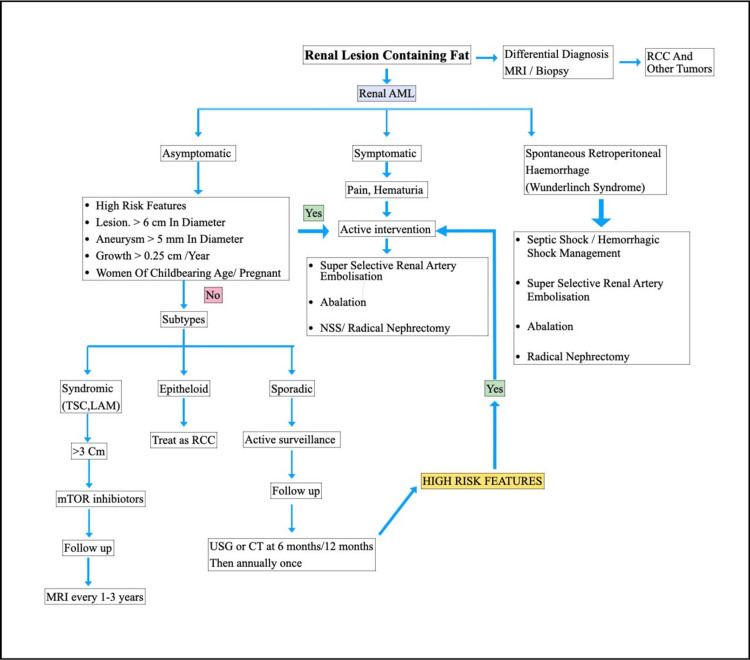
Management algorithm for renal angiomyolipoma RCC: Renal cell carcinoma; AML: angiomyolipoma; MRI: magnetic resonance imaging; NSS: nephron-sparing surgery; cm: centimeter; mm: millimeter

We use the following algorithm (Figure 4) to treat the patients of renal AML and tailor it according to the patients presentation and clinical findings [18]. The current guidelines of the European Association of Urology recommend intervention in well-selected cases, including symptomatic tumors, large lesions, the presence in women of childbearing age, and poor access to follow-up or emergency care [19]. A size threshold for treatment, however, remains controversial.

## Conclusions

WS usually has a fatal outcome, largely due to hypovolemic shock and delayed diagnosis. A high index of clinical suspicion is required to diagnose this condition, and early diagnosis is the key to a better prognosis. Subcapsular hematoma is usually self-limiting, and masterly inactivity is the key to renal salvageability in patients with hemodynamic stability. In patients with refractory bleeding, early nephrectomy after stabilization is lifesaving. Prompt diagnosis, appropriate treatment, adequate paramedical support, and multidisciplinary team involvement are the keys to success in managing patients with WS.
